# Effect of *N*,*N*′-Dicyclohexyldicarboxamide Homologues on the Crystallization and Properties of Isotactic Polypropylene

**DOI:** 10.1021/acsomega.1c00064

**Published:** 2021-03-23

**Authors:** Flóra Horváth, Levente Bihari, Dominika Bodrogi, Tibor Gombár, Bendegúz Hilt, Balázs Keszei, Tamás Krain, András Simon, Alfréd Menyhárd

**Affiliations:** † Department of Physical Chemistry and Materials Science, Faculty of Chemical Technology and Biotechnology, Budapest University of Technology and Economics, Műegyetem rkp. 3, Budapest 1111, Hungary; ‡ Department of Inorganic and Analytical Chemistry, Faculty of Chemical Technology and Biotechnology, Budapest University of Technology and Economics, Szt. Gellért tér 4, Budapest 1111, Hungary

## Abstract

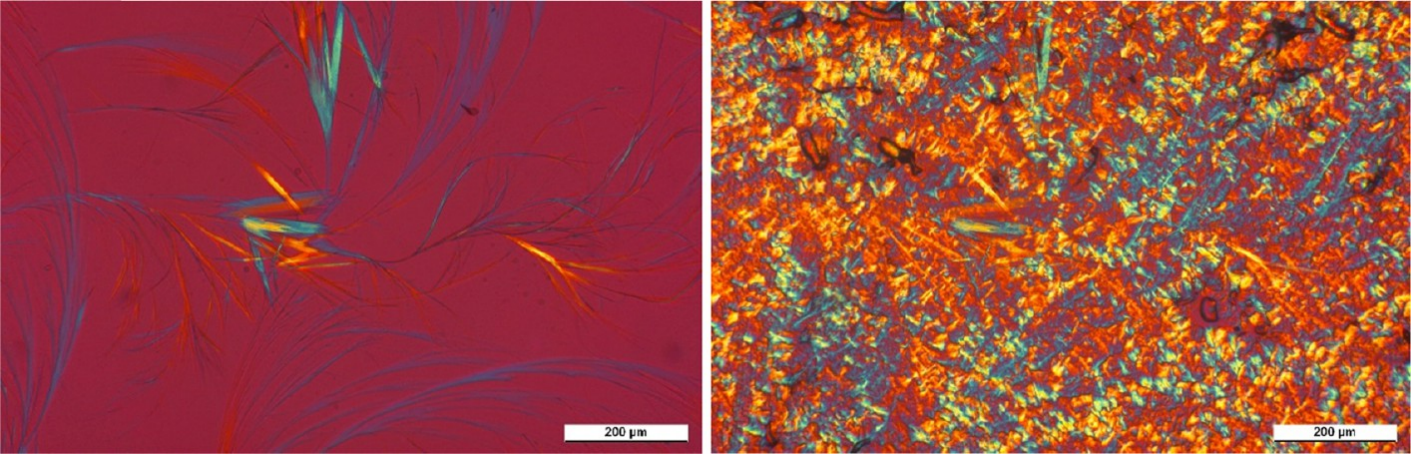

Application of nucleating agents is the most versatile and industrially applied way to manipulate the crystalline structure of isotactic polypropylene (iPP). Various materials possess a nucleating effect, but from the viewpoint of dispersibility, the partially soluble ones are the most advantageous. Our objective was to synthesize new *N*,*N*′-dicyclohexyldicarboxamide homologues and study their applicability as nucleating agents in iPP. Carbon-13 nuclear magnetic resonance (^13^C NMR) and infrared spectroscopy were used to prove that the synthesis reactions were successful. Thermal stability of the compounds was investigated with simultaneous thermal analysis. Nucleating efficiency and solubility were characterized by polarized light microscopy and differential scanning calorimetry. Polarized light microscopy was also applied to study the effect of novel additives on the morphology of iPP. The properties, important from the viewpoint of applicability, were also investigated. Tensile tests were performed to characterize the main mechanical properties, and standard haze measurements were performed to characterize optical properties. It can be concluded that the investigated compounds are partially soluble nucleating agents and influence the crystalline structure of iPP. Most of the studied compounds have a moderate nucleating efficiency, but a very interesting dendritic structure develops in their presence. Two of them proved to be non-selective β-nucleating agents, which result in a remarkable improvement of impact resistance and higher opacity.

## 1 Introduction

It is well-known how strongly the properties of a material depend on its structure. In the case of semicrystalline polymers, the crystalline structure developing in the material is fundamentally important from the viewpoint of the properties. Isotactic polypropylene (iPP) is a semicrystalline polymer with different polymorphic modifications,^1^ among which only the monoclinic (α) and trigonal (β)^2^ forms have industrial relevance, although during processing the other forms can develop in minor amounts, as well.^3^ The formation of these modifications requires special conditions. The orthorhombic γ-iPP was first prepared by Addink and Beintema^4^ from low-molecular-weight iPP at high pressure and in random copolymers with less regular chains, although this modification was observed in commercial injection-molded products as well due to the higher shearing conditions.^5^ Recently, Lotz et al.^6^ discovered another new form, ε-iPP. The thermodynamically stable polymorph is α-iPP; however, also the β-modification can develop in significant amounts during processing if favorable conditions are present. This can be advantageous from the viewpoint of applicability, due to the higher impact resistance of β-iPP compared to that of α-iPP.^2^ The crystalline structure formed in a product, besides conditions of crystallization (temperature, rate of cooling), depends on the presence of materials apart from the polymer. Nucleating agents are additives introduced in the polymer to manipulate the crystalline structure. There is a wide variety of nucleating agents with diverse chemical structures and characteristics. By the application of the proper nucleating agent, one can manipulate the crystalline structure of a polymer, thus preparing a product possessing the desired properties, like high transparency, improved mechanical properties, etc.

Several different materials can act as nucleating agents, from inorganic to organic, from small molecular to polymeric substances, as well. The main criteria of nucleating efficiency are (1) the presence of heterogeneous particles wettable by the polymer melt^7^ and (2) similarity between the crystalline structures of the particle and the nucleating agent.^8^ The more this latter requirement is fulfilled, the higher the nucleating efficiency of the additive is. From the viewpoint of dispersibility, two main ways of nucleation can be distinguished. Nonsoluble nucleating agents are particles dispersed in the polymer. These particles remain solid during the whole course of polymer processing; therefore, in the literature, they are generally referred to as “conventional” or “heterogeneous” nucleating agents. Various materials can be applied as nonsoluble nucleating agents, such as talc,^9−11^ sodium benzoate,^12,13^ sodium-2,2′-methylene-bis(4,6-di-*t*-butylphenyl) phosphate,^14^ calcium pimelate, and calcium suberate.^15,16^ In the case of these nucleating agents, the extent and the homogeneity of the dispersion of the additive powder have the uppermost importance. Another way is the application of materials that are partially soluble in the polymer melt in the temperature range of polymer processing. Owing to partial solubility, the dispersibility of these materials is much better compared to that of nonsoluble nucleating agents. However, as these materials are only slightly soluble in the polymer melt, they recrystallize during cooling, at a temperature higher than the crystallization temperature of the polymer; consequently, they are present as heterogeneous surfaces during the crystallization of the polymer. During their recrystallization, the molecules of the dissolved nucleating agent form uniformly and finely dispersed crystalline surfaces that are responsible for the efficient nucleation.

Nucleating agents can be classified also from the viewpoint of selectivity. Most of the nucleating agents are selective for α-iPP,^17^ which means that they nucleate only that modification. Relatively few nucleating agents are selective for the β-form of iPP, like calcium suberate and calcium pimelate.^16,18^ However, non-selective (or dual) nucleating agents also exist, and they nucleate both the α- and β-modifications of iPP. The first representative of dual nucleating agents was linear trans γ-quinacridone.^19^ In the case of the non-selective nucleating agents, the crystalline structure developing in the polymer depends on the thermal conditions. If the density of the β-nuclei is high enough, between 100 and 140 °C, the ratio of the β-modification will be large, like in the case of the commercial nucleating agent NJ Star NU 100^20^ and *N*,*N*′-dicyclohexyl-terephthalamide (DCHT).^21^ As a result of the high density of β-nuclei, crystallites of β-iPP can form, and owing to the favoring thermal conditions (between 100 and 140 °C, they grow faster than α-iPP), β-crystallites can grow. It was also observed that in this temperature range a special fanlike crystalline structure can form, in which the α-iPP crystallites are overgrown by the crystallites of β-iPP.^22^ The exact geometry can be calculated knowing the ratio of the growth rates of the two modifications.^23^ Another decisive factor is the concentration of the nucleating agent. As these nucleating agents are only partially soluble, if their concentration increases, the temperature at which they start to recrystallize from the polymer melt also increases. As a consequence, diverse crystalline structures can develop depending on the thermal conditions of crystallization.^20,24^

Although the nucleating ability of various types of compounds has been investigated, only one paper dedicated to the study of *N*,*N*′-dicyclohexyldicarboxamides exists in the literature.^25^ This work provides valuable information; however, measurements were restricted to the thermal characterization of samples nucleated by the investigated additives, and the detailed characterization of the additive as well as the investigation of the morphology of the nucleated samples was not discussed at all.

The main objective of our work was the thorough characterization of a family of *N*,*N*′-dicyclohexyldicarboxamides, from the viewpoint of applicability as nucleating agents in iPP. The members of the family differ from each other only in the number of methylene groups located between the amide groups. The nucleating efficiency of the compounds was determined using differential scanning calorimetry (DSC) and polarized light microscopy (PLM). Tensile and impact tests were also performed to study the effect of additives on the mechanical properties. The influence of the nucleating agents on the optical properties was characterized by the measurement of haze. Investigation of the crystalline structure developing in the presence of the novel nucleating agents is also aimed in the study.

## 2 Experimental Section

### 2.1 Materials

**Figure 1:**
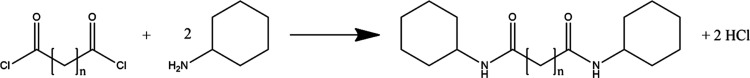
Schematic representation of the reaction and the general structure of the investigated compounds (*n* = 0–8).

The isotactic polypropylene used in the study was supplied by MOL Petrochemicals Hungary. It was a commercial homopolymer grade, TIPPLEN H 649 FH (MFR = 2.5 g/10 min at 230 °C, 2.16 kg). The nucleating agents were synthesized in our laboratory in the reaction of carbonyl dichlorides and cyclohexylamine (for the reaction scheme and general structure, see Figure 1), according to the procedure described in our previous paper.^21^ The types and the main characteristics of the raw materials used are given in Table 1.

**Table 1:** Materials Used in the Synthesis Reaction

material	molecular weight (g/mol)	role	supplier (purity)
oxalyl chloride	126.93	reagent	Sigma-Aldrich (98%)
malonyl chloride	140.98	reagent	TCI (>97.0%)
succinyl chloride	154.98	reagent	Sigma-Aldrich (95%)
glutaryl chloride	169.01	reagent	Sigma-Aldrich (97%)
adipoyl chloride	183.03	reagent	TCI (>98.0%)
pimeloyl chloride	197.06	reagent	Sigma-Aldrich (98%)
suberoyl chloride	211.09	reagent	TCI (>98.0%)
azelaic acid dichloride	225.11	reagent	TCI (>97.0%)
sebacoyl chloride	239.14	reagent	TCI (>95.0%)
cyclohexylamine	99.17	reagent	TCI (>99.0%)
diethyl ether	74.12	solvent	Molar Chemicals (a.r.)
triethylamine	101.19	acid scavenger	TCI (>99.0%)

The chemicals were purchased from the suppliers listed in Table 1 and were used without any further purification. The reactions were carried out in diethyl ether solvent, and triethylamine was used as the acid scavenger. The reactor, which was a four-neck round-bottom flask made of glass, was cooled with an ice bath to keep the temperature between 0 and 10 °C, which was continuously monitored. The reaction solution was stirred during the whole process under an inert argon atmosphere. First, the solvent, the acid scavenger, and the carbonyl dichloride were measured in the reactor. To make sure that the whole amount of the forming hydrogen chloride reacts with the acid scavenger, triethylamine was applied in an excess of 10% relative to the carbonyl dichloride. To avoid the formation of monosubstituted reaction products, the second reagent, cyclohexylamine, was applied in an excess of 20% relative to the carbonyl dichloride. As the reaction is exothermic, this second reagent was added drop by drop using a dropping funnel and the temperature was kept in the required range. After the reaction was completed, the product was yielded as a white or yellowish precipitate and was washed in multiple steps with distilled water and solutions of hydrogen chloride and sodium carbonate to remove residual nonreacted reagents.

### 2.2 Methods

The synthesis products were characterized by various methods. Their chemical structure was investigated by Fourier transform infrared (FTIR) spectroscopy and carbon-13 nuclear magnetic resonance (^13^C NMR) spectroscopy. ^13^C NMR spectra were recorded with a Bruker Avance 300 NMR spectrometer. In the FTIR investigations, a Bruker Tensor 27 instrument equipped with a DRIFT accessory was used, which enables the direct analysis of samples in the form of powders. The thermal properties important from the viewpoint of application (i.e., melting point and decomposition temperature) of the compounds were determined with simultaneous thermal analysis, using PerkinElmer STA 6000 equipment. Approximately 7–10 mg of the powders was placed in alumina crucibles and heated up from room temperature (50 °C) to 900 °C at a heating rate of 10 °C/min under a N_2_ atmosphere. Both the heat flow and the mass curves were registered and evaluated using Pyris software.

The following procedure was performed to investigate qualitatively the solubility and the nucleating effect of the synthesis products on iPP. A thin film of the neat polymer was prepared between two glass slides using a heated furnace, with accurate temperature regulation. The samples were heated to 220 °C and held there for 2 min to erase the thermal and mechanical prehistory. The samples were pressed gently by hand to make the thin polymer films; then, a weight was placed on the top of the sample cover to keep the thickness as small as possible during cooling. With this gentle technique, no further orientation and stress are introduced in the sample during cooling. After cooling down the film, the upper glass slide was removed, and a small amount of the powder was put on the polymer film. Then, the film was covered with a cover glass and inserted into a Mettler FP82HT hot stage, where it was subjected to the following thermal program. The thermal and mechanical prehistory of the sample was erased by holding the sample for 3 min at 220 °C. In the next step, the sample was cooled down to the crystallization temperature at a cooling rate of 10 °C/min. The sample was kept at this temperature until its crystallization was complete. During the process, the sample was examined by polarized light microscopy (PLM) using a Zeiss Axioskop optical microscope in which a λ-plate located diagonally between the crossed polarizers was used. The microscope was equipped with a Leica DFC 320 digital camera. Micrographs were taken in different stages of the cooling and the crystallization process.

The potential nucleating agents were first prepared in a smaller amount (around 5 g), and the powders were introduced in the polymer with a Brabender W 50 EHT internal mixer driven by a Brabender Plasti-Corder Lab-Station driving unit. The rotating speed was 50 rpm, and the additive was added to the polymer after its melting. The melt containing the additive was homogenized for a further 9 min at 190 °C. The samples prepared weighed approximately 41 g and contained the additives in different concentrations between 100 and 10 000 ppm. This smaller amount of nucleating agent was enough for the investigation of the nucleating efficiency of the additives in question.

The powders that proved to be somewhat efficient were synthesized in a larger amount (approximately 25 g) because the thorough characterization of the nucleated samples needed more material. The powders were introduced in the polymer by extrusion, using a Brabender DSK 42/7 twin-screw extruder, driven by a Brabender Plasti-Corder PLE 3000 driving unit. The rotating speed was 50 rpm, and the temperature profile was 210–220–230–230 °C from the hopper to the die. As a first step, masterbatches containing the compounds in higher amounts (10 000 or 5000 ppm) were extruded and then diluted with the neat polymer to prepare the samples containing the potential nucleating agents in different concentrations.

The nucleating efficiency of the synthesized products, as well as the crystallization and melting properties and the polymorphic composition of the nucleated samples, was investigated by differential scanning calorimetry, using a PerkinElmer Diamond DSC calorimeter. To erase the thermal and mechanical prehistory, samples of 3–5 mg were heated up to 220 °C and they were held at this temperature for 5 min. The samples were cooled down to 50 °C, kept at this temperature for 1 min, and then heated up to 200 °C. The applied heating (*V*_h_) and cooling rates (*V*_c_) were 10 °C/min during the whole process. In the case of the investigated additives, which proved to be effective for the β-modification of iPP, DSC measurements were performed again, applying another thermal program, proposed by Varga et al.^26^ Applying this method, the ratio of the α- and the β-modification can be determined from the data registered during the third heating run because the effect of βα-recrystallization is eliminated by not cooling the sample below the critical temperature (100 °C) in the second cooling run.

ISO 527 tensile bars were injection-molded from the nucleated samples using a Demag IntElect 50/330-100 machine. The temperature profile was 205–210–220–230 °C. The temperature of the mold was 40 °C. The holding pressure and time were 500 bar and 20 s, respectively. For the measurement of haze, 80 × 80 mm^2^ samples with 1 mm thickness were also injection-molded. The temperature profile and the mold temperature were the same as in the case of tensile bars, the holding pressure was 660 bar, and the holding time was 10 s. Before testing mechanical and optical properties of the specimens, at least 1 week was allowed pass to ensure that secondary crystallization was complete.

Mechanical tests were performed using the injection-molded ISO 527 bars. Tensile tests were performed using an Instron 5566 apparatus with a gauge length of 115 mm. In the first step of the measurement, up to 0.3% elongation, the cross-head speed was 0.5 mm/min. The tensile modulus of the samples was determined from the data recorded in this elongation range. In the next step of the measurement, the cross-head speed was 50 mm/min, and the deformation lasted until the sample broke. Specimens for impact tests were cut from the ISO 527 bars also, from the center part where the cross section is 10 × 4 mm^2^. Impact tests were carried out using a Ceast Resil 5.5-type machine equipped with a 1 J hammer, according to the Charpy ISO 179-1 standard. Both tensile and impact tests were performed at 23 °C and 50% relative humidity.

Measurement of haze on the injection-molded plates was carried out using a Hunterlab ColorQuest XE machine following standard ASTM D-1003-95.

The structure developed in the nucleated samples was also investigated by polarized light microscopy (PLM), according to the procedure described above.

## 3 Results and Discussion

### 3.1 Structure of the Synthesis Products

When designing this family of nucleating agents, we considered some critical aspects. The peripheral cyclohexyl group appears in other efficient nucleating agents, like *N*,*N*′-dicyclohexyl-terephthalamide^21^ and *N*,*N*′-dicyclohexyl-2,6-naphthalenedicarboxamide;^20^ thus, we supposed that it enhances the nucleating efficiency in some way. The amide functional groups have a dual role. On the one hand, they guarantee thermal stability of the substance, and on the other hand, they promote one-dimensional crystal growth of the compound, leading to a higher surface-to-volume ratio.^27^ The idea of inserting an aliphatic chain between the amide groups arose because the same unit appears in the highly efficient β-nucleating agents, like calcium suberate and calcium pimelate.^15^Table 2 contains all of the compounds we aimed to prepare. Unfortunately, the preparation of two materials (DCHMaA and DCHAdA) was unsuccessful because of unknown reasons.

**Table 2:** Names and Abbreviations of *N*,*N*′-Dicyclohexylcarboxamide Compounds

number of methylene units^a^	name	abbreviation of the name
0	*N*,*N*′-dicyclohexyloxamide	DCHOxA
1	*N*,*N*′-dicyclohexylmalonamide	DCHMaA
2	*N*,*N*′-dicyclohexylsuccinylamide	DCHScA
3	*N*,*N*′-dicyclohexylglutarylamide	DCHGlA
4	*N*,*N*′-dicyclohexyladipoylamide	DCHAdA
5	*N*,*N*′-dicyclohexylpimeloylamide	DCHPiA
6	*N*,*N*′-dicyclohexylsuberoylamide	DCHSubA
7	*N*,*N*′-dicyclohexylnonanediamide	DCHAzA
8	*N*,*N*′-dicyclohexylsebacoylamide	DCHSeA

a See Figure 1.

**Figure 2:**
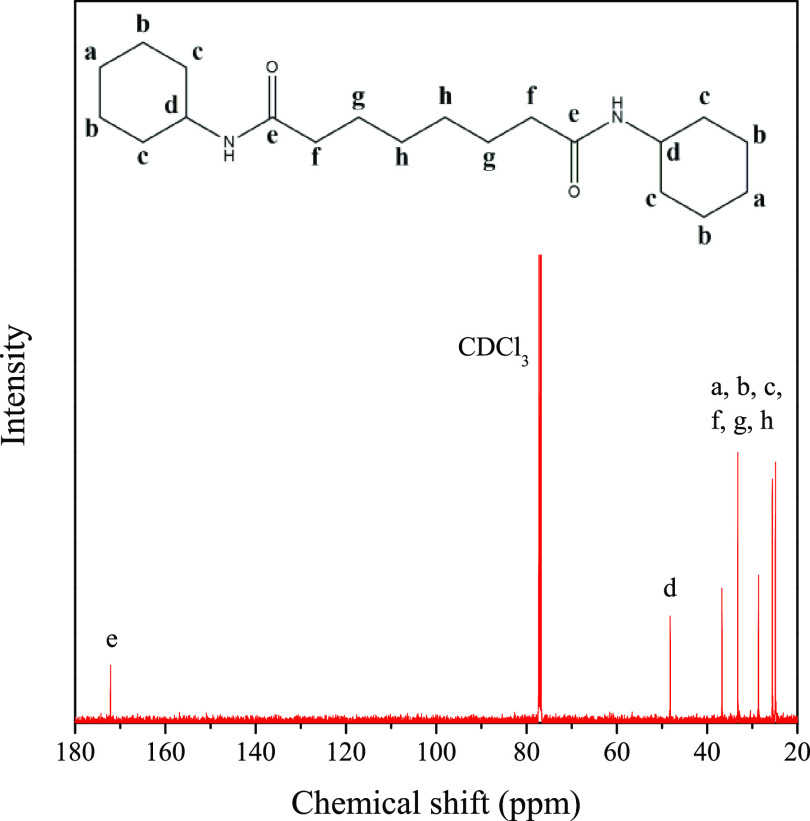
Structure and ^13^C NMR spectrum of DCHSubA.

We used ^13^C NMR spectroscopy and FTIR spectroscopy to make sure that in the reactions the expected products had been formed. In Figure 2, we present the ^13^C NMR spectrum of *N*,*N*′-dicyclohexylsuberoylamide (DCHSubA). On the spectrum, the most important peak is the one located at a chemical shift of 172 ppm. This peak corresponds to the carbon atom of the amide bond, marked with the letter e. Generally, the chemical shift of a carbon atom in a C=O bond is located between 150 and 220 ppm. A narrower range, 165–175 ppm, corresponds to amides or esters; however, as in this reaction the formation of esters cannot occur, this peak proves the presence of amide groups. The number of peaks together with the lack of a peak between 175 and 185 ppm proves that the product is symmetric, i.e., no half-substituted byproduct is formed. If one end of the suberoyl chloride molecule had not reacted with cyclohexylamine, it would have reacted with water when purifying the product; thus, a peak between 175 and 185 ppm, corresponding to the as-formed carboxyl group, would have appeared on the spectrum. The peak at 48 ppm belongs to the carbon atom marked with the letter d since the peak of a carbon atom in a C–N bond has a peak located around 50 ppm. The other peaks in the lower chemical shift area belong to the carbon atoms in the methylene groups.

**Figure 3:**
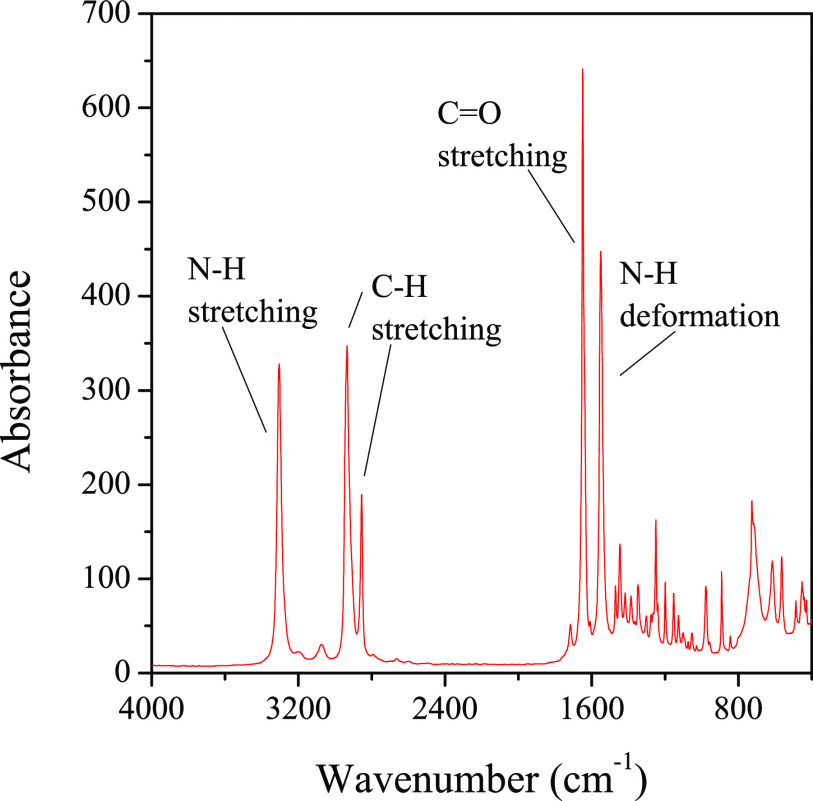
FTIR spectrum of DCHSubA.

The FTIR spectrum of DCHSubA is presented in Figure 3. The peak located at 3305 cm^–1^ belongs to the stretching of the N–H bond in the amide group. Two sharp peaks, which are located in the 2950–2850 cm^–1^ wavenumber range, correspond to the stretching of the C–H bonds in the methylene groups. The strong sharp peak located at 1650 cm^–1^ belongs to the stretching of the C=O bond in the amide group. The formation of the amide group is corroborated also by the presence of a peak located around 1550 cm^–1^, which belongs to the deformation vibration of the N–H bond in the amide group. Finally, the weak signal located at 1447 cm^–1^ belongs to the deformation vibration of the C–H bond, which is generally located between 1470 and 1450 cm^–1^.

The presence of the starting compounds can be excluded, as well. Primary amines, like cyclohexylamine, have a double signal in the range of 3500–3300 cm^–1^, which corresponds to the symmetric and antisymmetric stretchings of the N–H bond. Another signal, located between 1650 and 1580 cm^–1^, is also characteristic of the primary amines, and it corresponds to the deformation vibration of the N–H bond of amines. Neither this latter nor the previously mentioned double signal is present in our spectrum, which means that the product does not contain cyclohexylamine. The absence of suberic acid, which can form in the purifying step from the suberoyl chloride reacted with water, can also be excluded. A strong and broad peak in the range of 3300 and 2500 cm^–1^, corresponding to the stretching of the O–H bond, and a strong and sharp signal between 1780 and 1710 cm^–1^, belonging to the stretching of the C=O bond, are generally characteristic of carboxylic acids. Both signals are missing from our spectrum, which proves the absence of carboxylic acids.

We demonstrated our results presenting the spectra of DCHSubA as an example because of the large amount of data, but the results of ^13^C NMR and FTIR spectroscopy investigations were similar for all of our materials, as they differ from each other only in the number of methylene groups. Nevertheless, ^13^C NMR and FTIR spectra are given as Supporting Information. The results of ^13^C NMR and FTIR investigations prove that in the synthesis reactions the expected products formed.

### 3.2 Thermal Properties of the Synthesized Products

**Figure 4:**
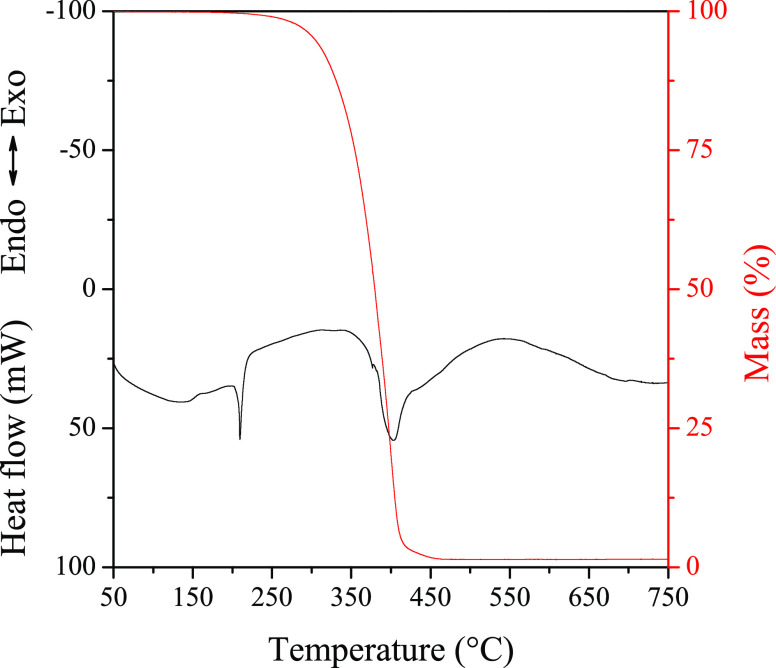
Thermal stability and behavior of DCHSubA.

The melting behavior and the decomposition temperature of the synthesized materials are discussed in the following. Beck^7^ specified some requirements for nucleating agents, in his article published in 1967. Among these, we have to mention two: (1) the melting point of a nucleating agent has to be higher than that of the polymer and (2) a nucleating agent has to be inert and chemically stable. By the determination of the melting point and the decomposition temperature, the fulfillment of these criteria can be verified. In Figure 4, we present the heat flow and the mass as functions of temperature in the case of DCHSubA. In the heat flow vs temperature curve, an endothermic peak appears around 210 °C, which corresponds to the melting point of the material. Another endothermic peak appears at 390 °C, which belongs to the decomposition of the material, and it is accompanied by a pronounced mass loss event, as well. The melting point and decomposition temperature values for all synthesized products are collected in Table 3. We selected the temperature that belongs to the 5% mass loss as the characteristic decomposition temperature.

**Table 3:** Melting Point and Decomposition Temperature Values of the Synthesized Products

material (number of methylene units)	melting point (°C)	decomposition temperature (°C)
DCHOxA (0)	decomposes without melting	187.51
DCHScA (2)	241.95	247.53
DCHGlA (3)	208.63	252.13
DCHPiA (5)	185.68	278.57
DCHSubA (6)	207.95	303.54
DCHAzA (7)	169.85	277.44
DCHSeA (8)	185.53	289.30

**Figure 5:**
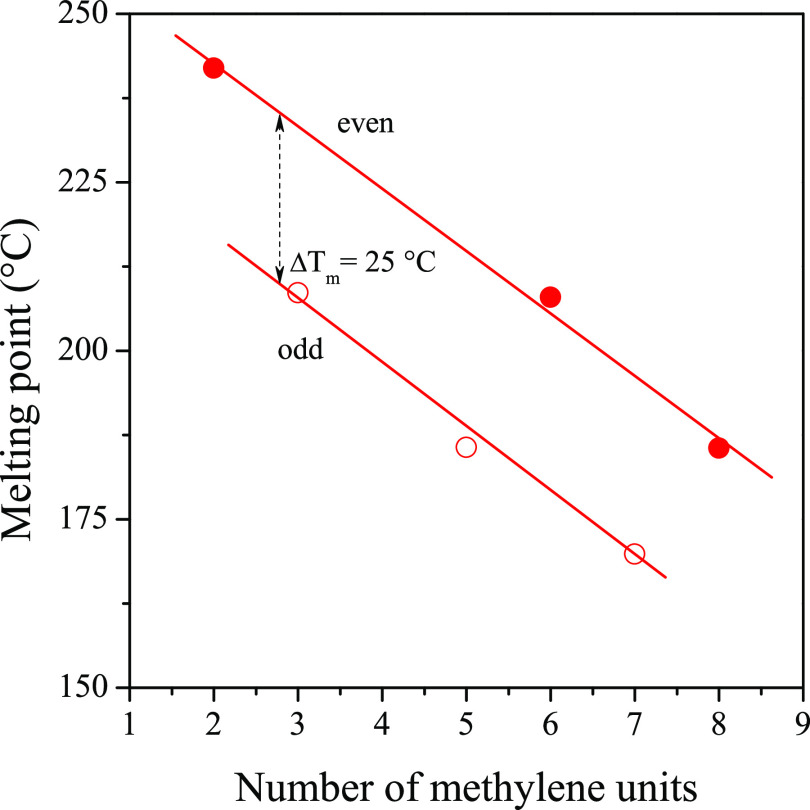
Melting point of the materials as a function of the number of methylene groups.

Based on the results, we can establish that most of the investigated materials fulfill the abovementioned requirements. DCHOxA undergoes decomposition without melting, below the temperature of processing; therefore, it cannot be applied as a nucleating agent in iPP. The industrial application of DCHScA and DCHGlA is also questionable since the melt temperature is often higher than 230 °C during processing. All of the other compounds have higher decomposition temperatures than 260 °C; thus, they can be applied even in industrial processes without the danger of decomposition. The decomposition temperature values increase with the number of methylene groups, i.e., with the molecular weight. In Figure 5, we present the dependence of the melting point on the number of methylene groups. An interesting trend can be recognized: molecules in which the number of methylene groups is odd tend to have a lower melting point by approximately 25 °C, compared to molecules with an even number of methylene groups. Furthermore, the melting point decreases with increasing molecular weight. We have to note here that all melting points are higher than the crystallization temperature range of the iPP, which is essential from the point of view of nucleating ability.

### 3.3 Nucleating Efficiency

**Figure 6:**
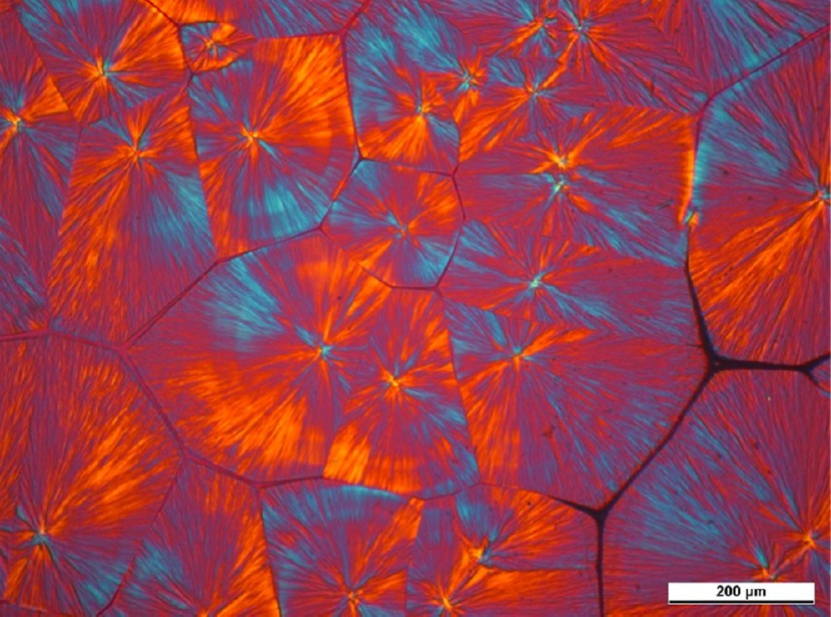
Crystalline structure of a non-nucleated iPP sample, developed during isothermal crystallization at 135 °C.

The first step was the qualitative investigation of the solubility and the nucleating efficiency of the powders on iPP films, using the PLM technique. We present the PLM micrograph of a neat iPP film in Figure 6, to make the differences between the neat and nucleated samples more clearly observable. Large spherulites of the α-modification of iPP formed, with clear grain boundaries. At the temperature of isothermal crystallization (*T*_c_ = 135 °C), the sample needed nearly 4 h to completely crystallize.

**Figure 7:**
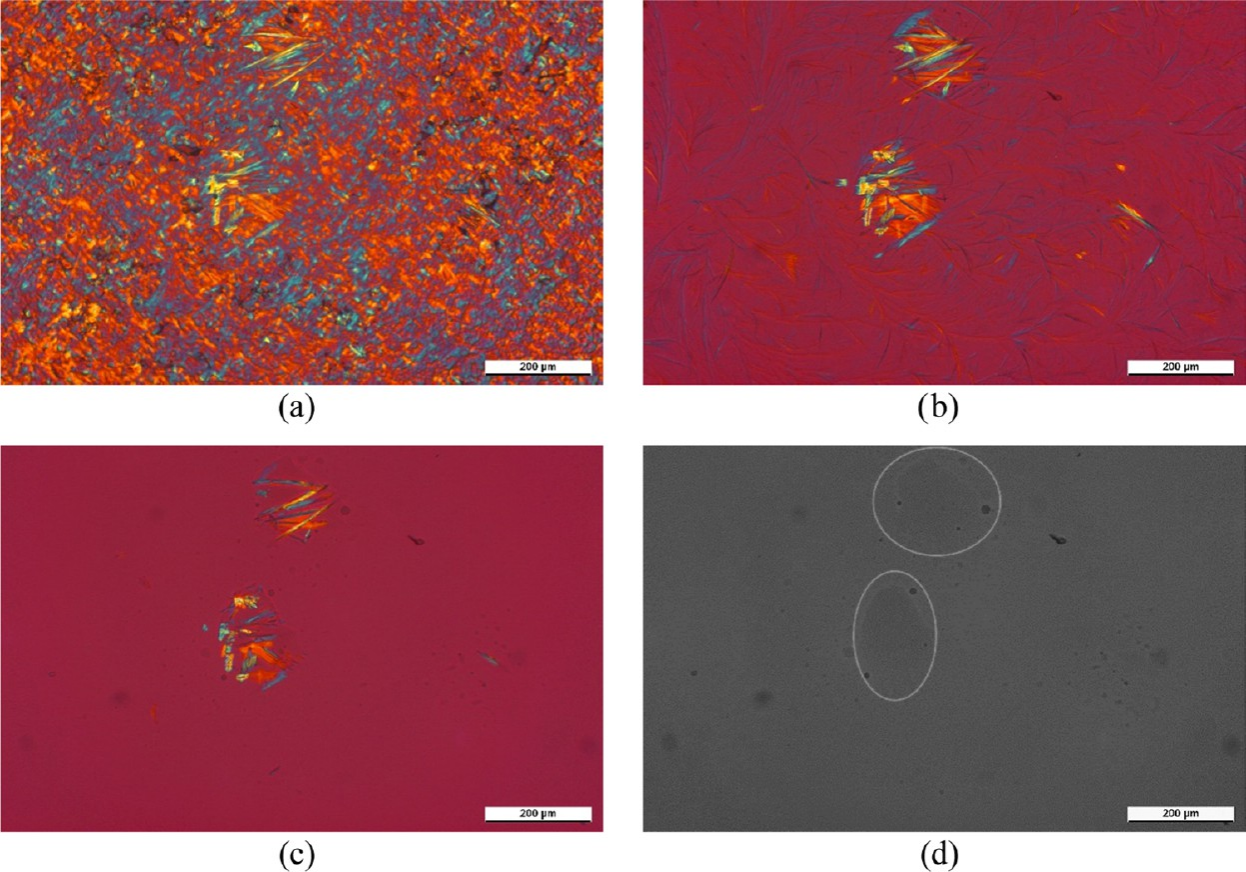
Qualitative investigation of solubility of DCHSubA in iPP. Micrographs were taken in the course of heating at (a) 106 °C, (b) 166 °C, (c) 200 °C; *t* = 0 min, and (d) 200 °C; *t* = 15 min.

**Figure 8:**
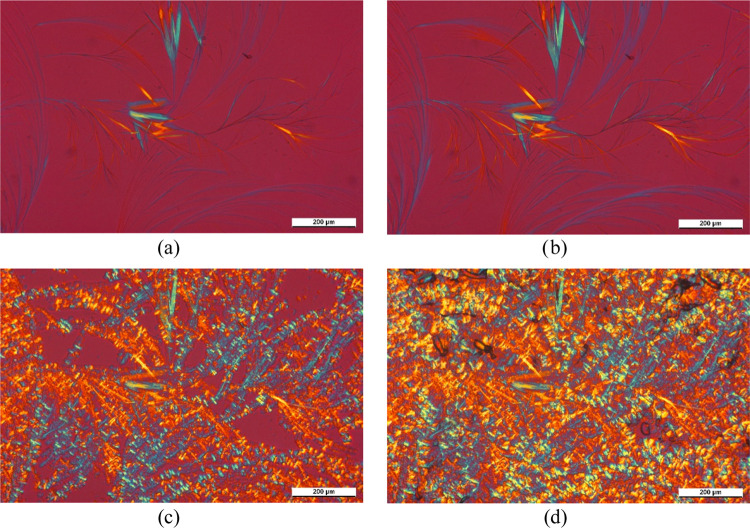
Qualitative investigation of the nucleating efficiency of DCHSubA in iPP. Micrographs were taken in the course of cooling at (a) 180 °C, (b) 130 °C, (c) 125 °C; *t*_c_ = 0 min, and (d) 125 °C; *t*_c_ = 90 s.

The micrographs taken during the study of the effect of DCHSubA are given in Figures 7 and 8. The pictures of Figure 7 were taken during the heating course when the sample was heated up to 200 °C. Figure 7a shows the sample at 106 °C. At this temperature, the crystalline structure of the neat polymer can be clearly seen, and also the lathlike crystals of DCHSubA are visible. At 166 °C (Figure 7b), the polymer is completely melted; however, a network of fibrils is observable. As the temperature increases, this network disappears, and the size of the DCHSubA crystals also decreases (Figure 7c). After holding the sample at 200 °C for 15 min, the crystals completely disappear, although drops remain at their place (Figure 7d). We have to note that we changed the colors of this picture to make the droplets of DCHSubA more visible. Considering that the highest applied temperature (200 °C) is below the melting temperature of DCHSubA (208 °C), we can declare that this material is partially soluble in iPP melt. This is also corroborated by the fact that above the melting temperature of the polymer a fibrillary network formed, which undoubtedly has to be built up by the dissolved and recrystallized DCHSubA.

In the next series of pictures (Figure 8), the micrographs taken during the cooling run and the following isothermal crystallization at 125 °C are presented. At 180 °C, DCHSubA starts to recrystallize, mainly in the area where the droplets remained, i.e., where its local concentration in the polymer melt is higher (Figure 8a). At 130 °C, the fibrillary network of DCHSubA is more clearly visible (Figure 8b). At 125 °C (Figure 8c,d), crystallization of iPP rapidly occurs. Comparing Figure 6 and Figure 8d, it is clear that the crystalline structure of the polymer is different in the presence of DCHSubA; therefore, it has a nucleating effect in iPP. The stronger birefringence of the sample (Figure 8d) implies the formation of β-iPP. The micrographs presented in Figures 7 and 8 clearly prove that DCHSubA is partially soluble in the iPP melt and, after its crystallization, influences the crystallization process of the polymer.

**Figure 9:**
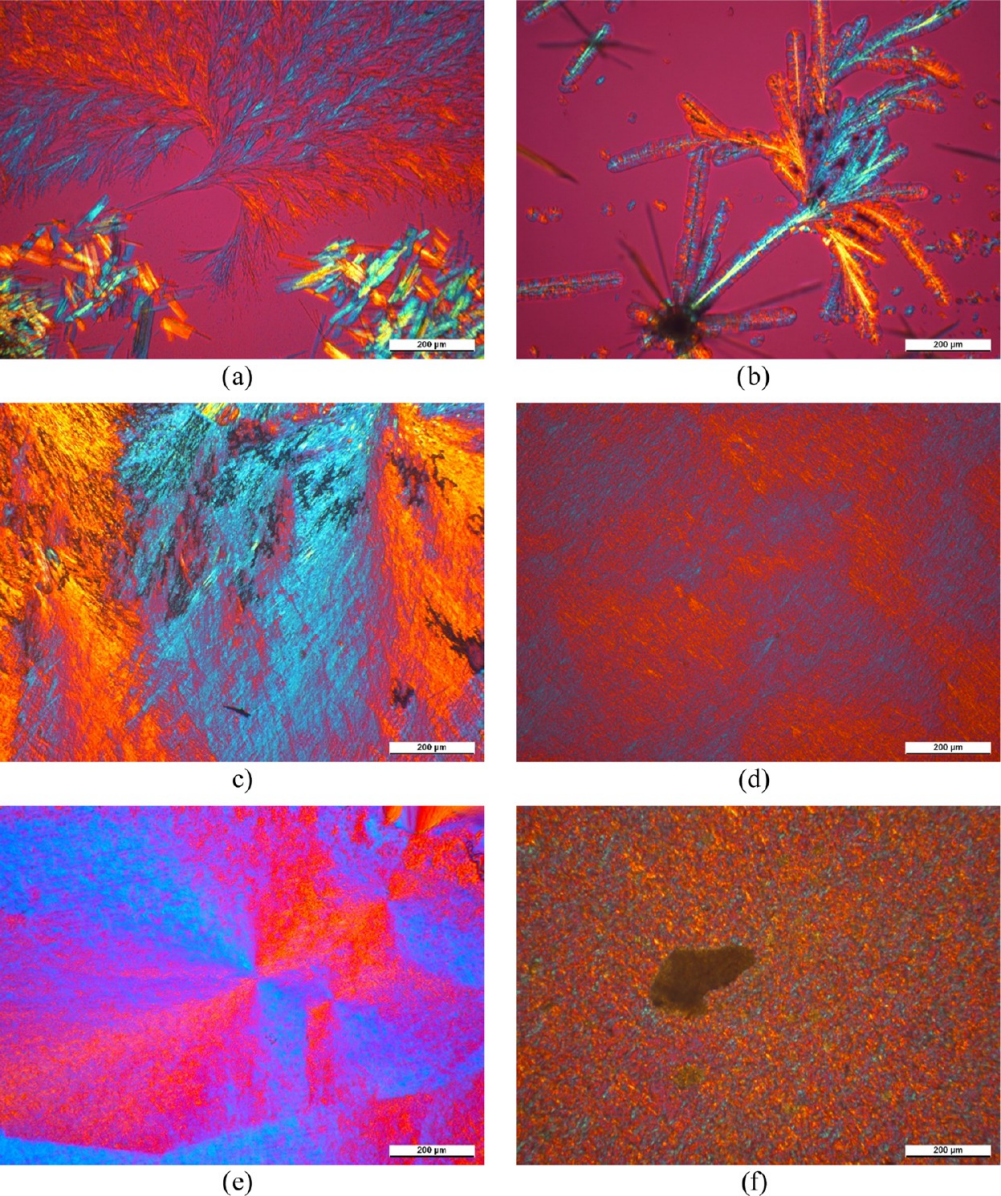
Crystalline structure of the polymer after isothermal crystallization in the presence of (a) DCHOxA (*T*_c_ = 135 °C, *t*_c_ = 1 min), (b) DCHScA (*T*_c_ = 130 °C, *t*_c_ = 1 min), (c) DCHGlA (*T*_c_ = 135 °C, *t*_c_ = 5 min), (d) DCHPiA (*T*_c_ = 125 °C, *t*_c_ = 1 min), (e) DCHAzA (*T*_c_ = 130 °C, *t*_c_ = 1 min), and (f) DCHSeA (*T*_c_ = 135 °C, *t*_c_ = 1 min).

The crystalline structure formed in the presence of each investigated compound is presented in the micrographs of Figure 9. Comparing these micrographs to the one representing the neat polymer (Figure 6), we can establish that all of them influence the crystalline structure of iPP, and they are partially soluble in the polymer melt. Additionally, the branching, dendritic morphology of the recrystallized compounds, is more or less similar. Despite its low decomposition temperature, this investigation was performed also with DCHOxA (Figure 9a) to find out if it has a modifying effect on the crystalline structure of the polymer. Like the other compounds, DCHOxA is also soluble in the iPP melt, and it recrystallizes in a similar dendritic structure, indicating its nucleating effect. However, its insufficient thermal stability makes it unusable as a nucleating agent in industrial practice; thus, no other investigations were performed on this material.

**Figure 10:**
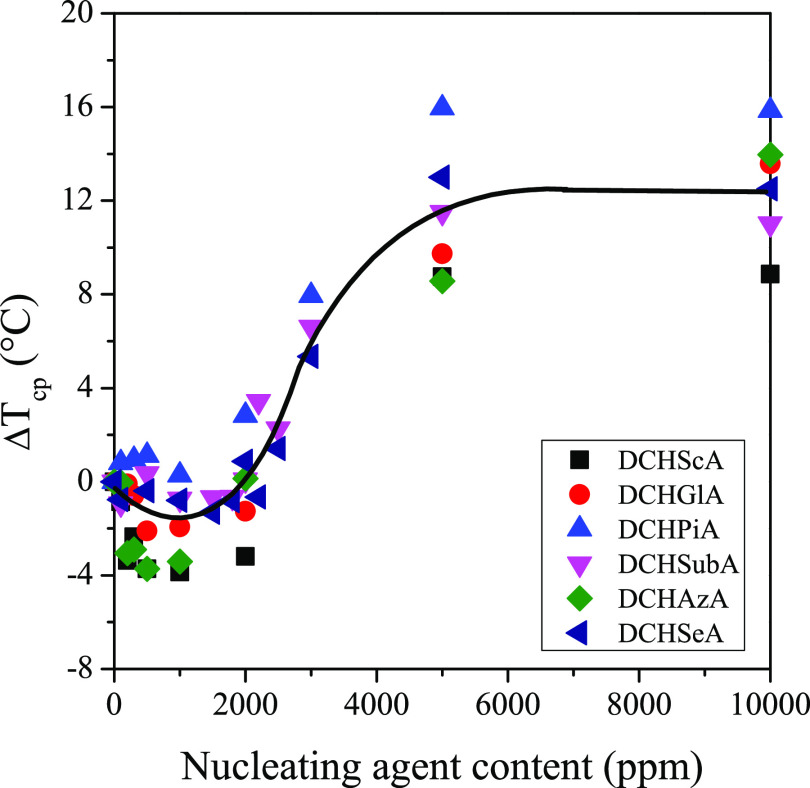
Shift in the crystallization peak temperatures (Δ*T*_cp_) as a function of the amount of potential nucleating agents.

Besides the qualitative investigation, DSC measurements were carried out on the nucleated samples to study the nucleating efficiency of the compounds quantitatively. We characterized our potential nucleating agents with the shift in the crystallization peak temperatures compared to the neat polymer (Δ*T*_cp_), instead of the absolute value of the crystallization peak temperature (*T*_cp_). As our experiments required a large amount of the iPP homopolymer, it was inevitable to use polymers produced in different batches. Therefore, there are small differences between the properties of the different batches. In Figure 10, we show the Δ*T*_cp_ values as a function of the amount of potential nucleating agents.

With the curved line, we intend to make the trend of the data points more clearly visible. The same tendency can be observed in the case of all compounds. Below 2000 ppm, Δ*T*_cp_ values are negative, i.e., the crystallization peak temperature of the nucleated samples is lower than that of the neat polymer. This phenomenon is described in the literature by Kristiansen et al.^24^ in the case of organogelators, a special family of partially soluble nucleating agents. In the range of smaller nucleating agent contents, under cooling the crystallization of the additive does not occur, it remains dissolved in the polymer. Besides the effect described in the mentioned literature, another phenomenon can play a role in the decrease of *T*_cp_ values. The dissolved molecules of the nucleating agent have no effect on the developing crystalline structure of the polymer, but they decrease the mobility of the macromolecules, thus hindering them in the crystallization process, which explains the decrease of *T*_cp_ values. Crystallization peak temperatures increase significantly above 2000 ppm and reach a plateau around 5000 ppm. This trend is characteristic of the partially soluble nucleating agents; thus, it can be concluded that the studied compounds possess a nucleating effect in iPP and belong to the family of partially soluble nucleating agents.

**Figure 11:**
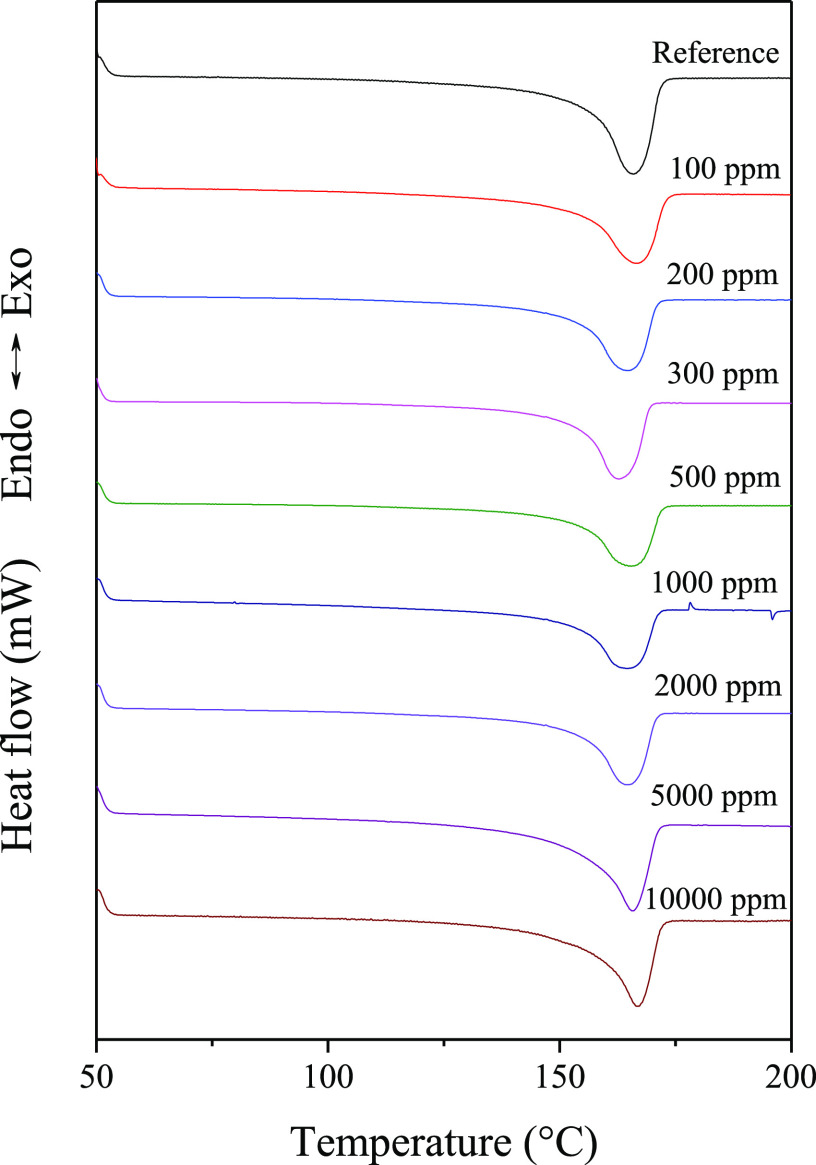
Melting curves of iPP samples nucleated with DCHScA.

In Figure 11, we present the DSC curves registered during the second heating run of nucleated samples containing DCHScA, as an example of the compounds that proved to have a nucleating effect in iPP. One peak appears on each curve, with minima around 165 °C, which is the melting temperature of α-iPP. On increasing the nucleating agent concentration, the temperature range of melting becomes somewhat narrower, while the peak temperature of melting slightly increases. This implies that in the presence of the nucleating agent, owing to the elevated crystallization temperature, a more perfect lamellar structure can develop in the crystalline phase of iPP.

**Figure 12:**
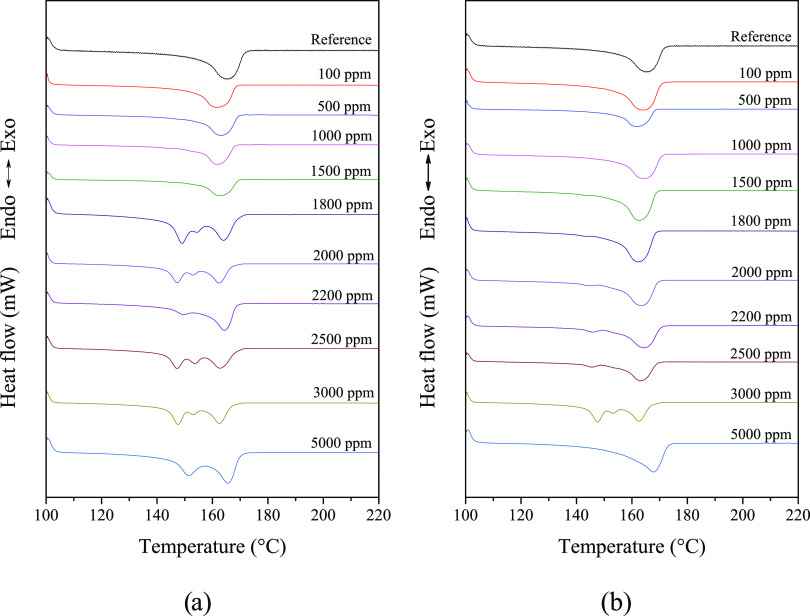
Melting curves of iPP samples in the presence of (a) DCHSubA and (b) DCHSeA.

Dual nucleating ability was observed in the case of two investigated compounds, namely, DCHSubA and DCHSeA. Figure 12 shows the melting curves of iPP samples nucleated with these two nucleating agents. Below 2000 ppm (i.e., the approximate solubility limit of the nucleating agents), the additives have no nucleating effect on β-iPP. We can conclude that the nucleating efficiency of DCHSubA is a little bit higher than that of DCHSeA, as the second melting peak around 155 °C, characteristic of β-iPP, appears at a lower concentration of 1800 ppm, while a very small second peak appears only at 2200 ppm in the presence of DCHSeA.

**Figure 13:**
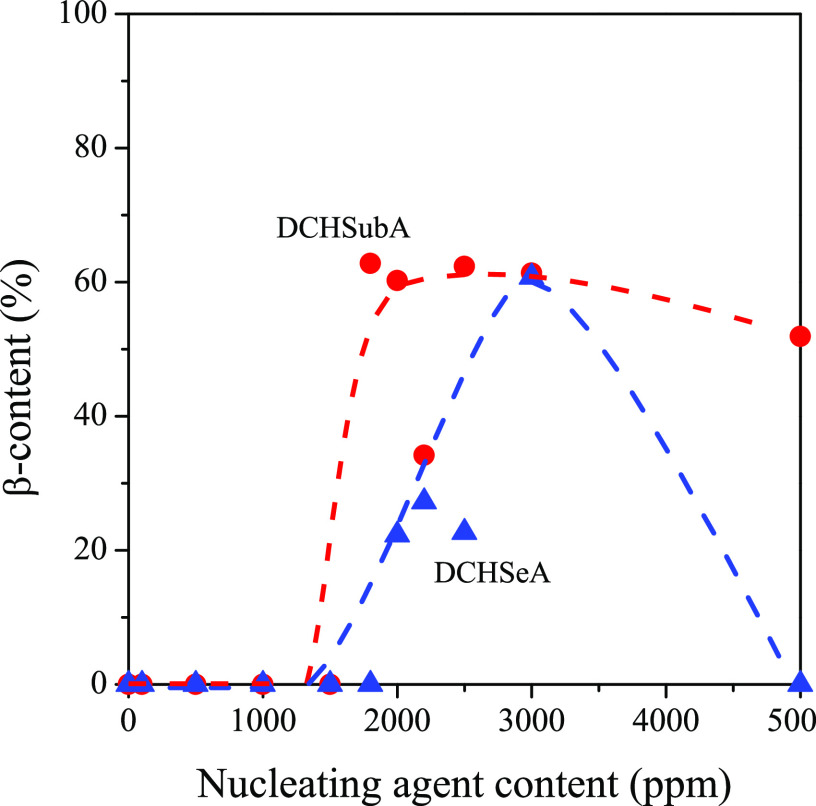
Ratio of β-iPP in the crystalline phase in the presence of DCHSubA (red solid circle) and DCHSeA (blue solid triangle) as a function of the concentration of the nucleating agents.

To explain this interesting effect, we have to consider that the developing crystalline structure depends on three factors: the rate of polymer crystallization, the growth rates of polymorphic modifications of iPP, and the recrystallization of the nucleating agent. These factors are influenced by the temperature and the nucleating agent content. In the case of DCHSubA, the solubility limit is around 1500–1800 ppm, which means that above this concentration range the nucleating agent recrystallizes at a higher temperature than the polymer; thus, it has a nucleating effect. In the presence of 1500 ppm DCHSubA, the polymer starts to crystallize prior to the crystallization of the nucleating agent, and hence the additive is not effective in this concentration. When the nucleating agent is around 1800 ppm, the crystallization of the polymer and the recrystallization of the nucleating agent take place simultaneously; consequently, the *T*_cp_ value does not increase, but the β-modification of iPP appears. To make the relative amount of the two polymorphic modifications more visible, we show the ratio of the β-modification (β-content) as a function of the nucleating agent content, in the cases of DCHSubA and DCHSeA (Figure 13). It is absolutely clear that these nucleating agents are not selective to the β-modification of iPP because the β-content shows a maximum with increasing nucleating agent content. Both the final polymorphic composition and the developing crystalline structure depend on the nucleating agent concentration and the temperature. These conditions determine the dissolution and recrystallization of the nucleating agent, as well as the crystallization of the polymer and the growth rate of the modifications. The maximum of the β-content is around the solubility limit of the nucleating agent (2000 ppm), similar to materials previously investigated.^20,21^ Applying the nucleating agents in this concentration, we suppose that their whole amount dissolves in the polymer melt and that most of it recrystallizes prior to the crystallization of the polymer. On further increasing the concentration, the ratio of the dissolved and recrystallized nucleating agent decreases; thus, the amount of α-iPP increases.

### 3.4 Crystalline Structure Developing in the Nucleated Polymer

**Figure 14:**
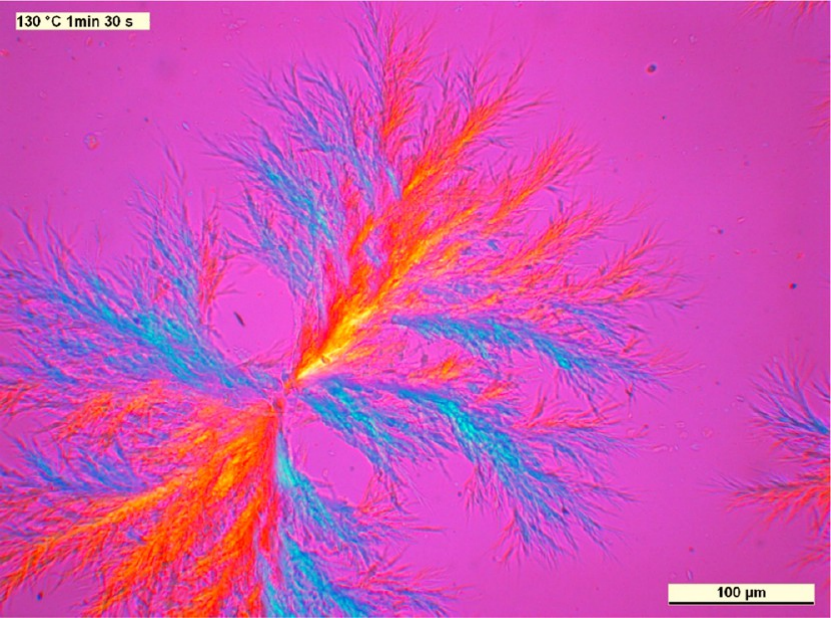
PLM micrograph taken during isothermal crystallization of iPP containing 2000 ppm DCHScA, as an example of the compounds having α-nucleating effects (*T*_c_ = 130 °C, *t*_c_ = 90 s).

**Figure 15:**
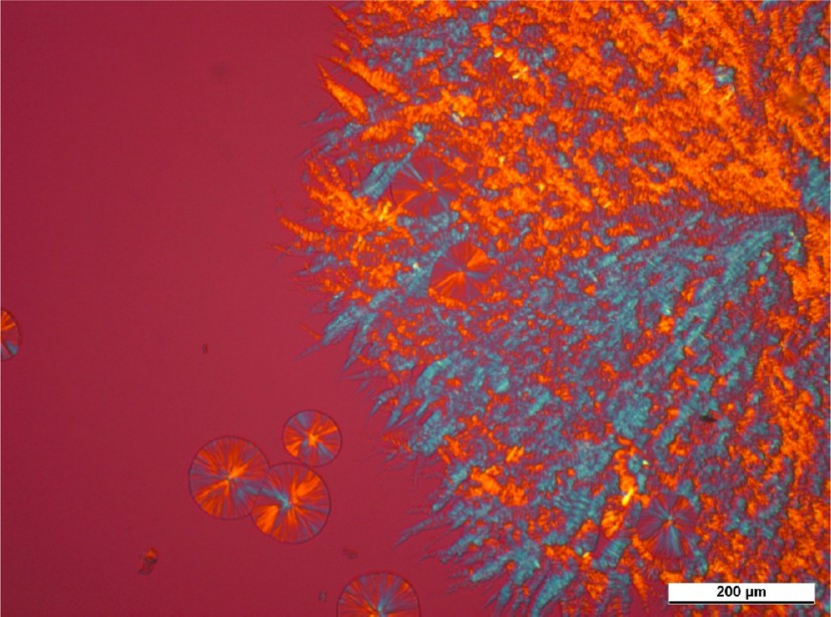
PLM micrograph taken during isothermal crystallization of iPP containing 2500 ppm DCHSubA (*T*_c_ = 135 °C, *t*_c_ = 30 min).

**Figure 16:**
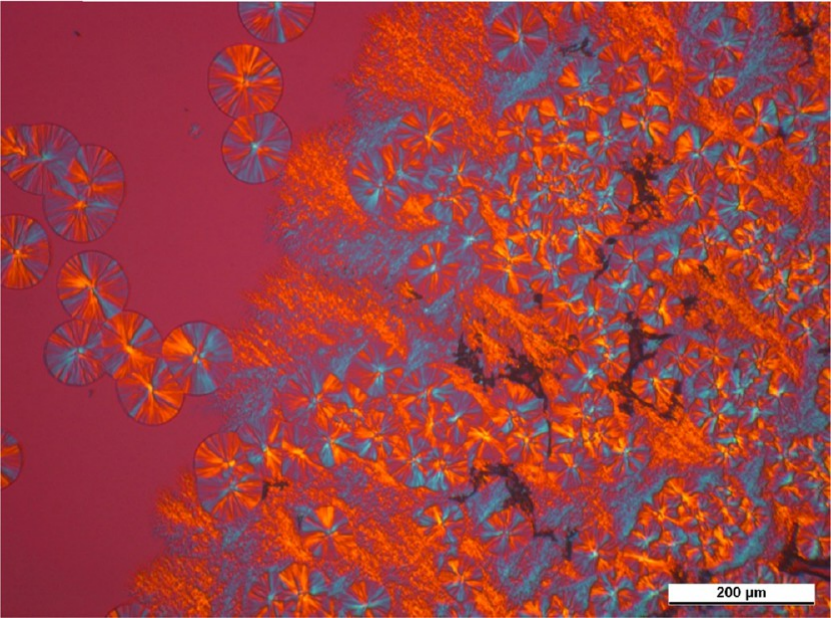
PLM micrograph taken during isothermal crystallization of iPP containing 2500 ppm DCHSeA (*T*_c_ = 130 °C, *t*_c_ = 9 min).

We show micrographs taken on samples containing the nucleating agents in concentrations near the solubility limit in Figures 14–16. To make the structure more observable, we chose pictures that were taken before the crystallization of the polymer ends.

A unique crystalline structure develops in the polymer in the presence of the investigated compounds. The nucleating agents recrystallize in the form of fibrillary crystals having a dendritic structure. The surface of these crystals serves as nucleation sites for the polymer; thus, the developing crystalline structure is neither microspherulitic nor microcrystalline. On the other hand, some spherulites are also discernible, which form in the early stage of cooling, when the dissolved amount of nucleating agent is below the saturation point, and the recrystallization has not started.

### 3.5 Mechanical Properties of Nucleated Samples

**Figure 17:**
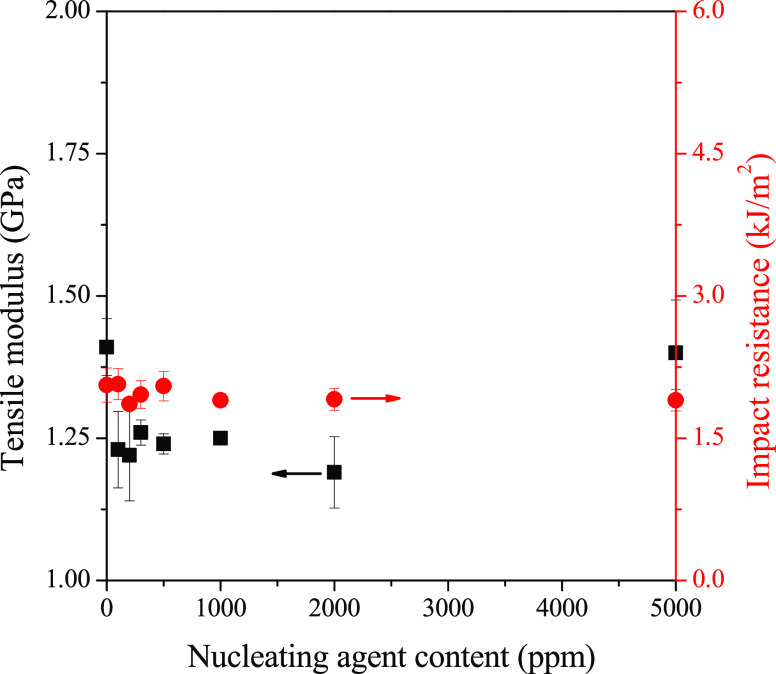
Tensile modulus and impact resistance of samples containing DCHScA as an example for the α-nucleating agents.

As there is always a correlation between structure and properties, it is important to study how the changes in the crystalline structure influence the properties. We characterized mechanical properties of the nucleated samples by their tensile modulus and impact resistance values. We show these properties as a function of the nucleating agent content, in the case of DCHScA in Figure 17. We can conclude that there is no significant change in the tensile modulus and the impact resistance with increasing nucleating agent content, which corresponds to the DSC data, proving that only the α-modification of iPP formed. We have to note here that the results were quite similar for the other investigated compounds, having no β-nucleating effect.

**Figure 18:**
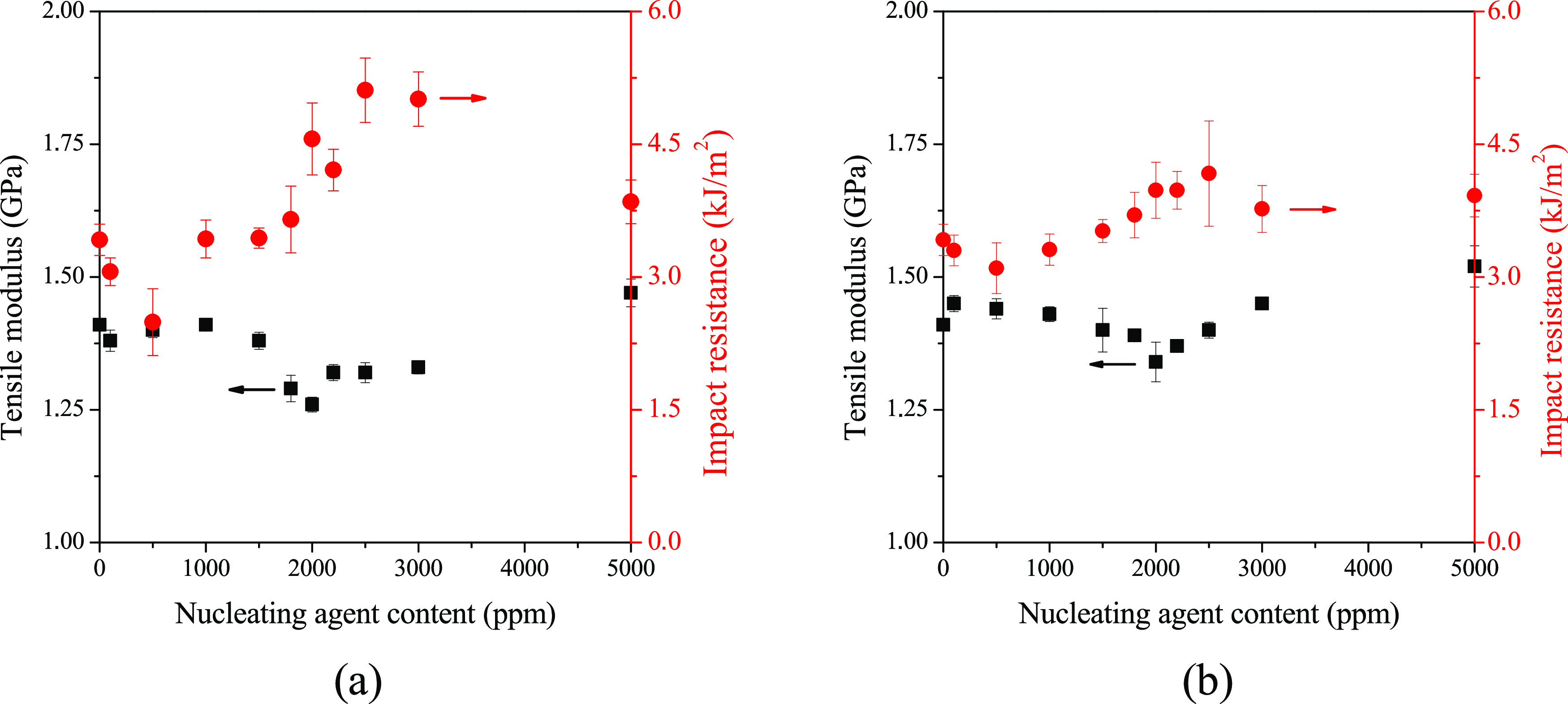
Tensile modulus and Charpy impact resistance of samples containing (a) DCHSubA and (b) DCHSeA in different concentrations.

In contrast, both stiffness and impact resistance changed in the cases of DCHSubA and DCHSeA, as can be seen in Figure 18. In the presence of these compounds, mechanical properties undoubtedly change above their solubility limit. The changes are more expressed in the case of DCHSubA, which correlates with our previously presented results, showing that DCHSubA is more efficient than DCHSeA. Additionally, Charpy impact resistance starts to increase around the solubility limit of the nucleating agents, and after a maximum, it decreases with increasing concentration, i.e., an optimal concentration of the nucleating agents can be determined. As tensile modulus and impact resistance are usually inversely proportional, tensile modulus has a minimum value around the concentration where impact resistance reaches a maximum.

### 3.6 Optical Properties of Nucleated Samples

**Figure 19:**
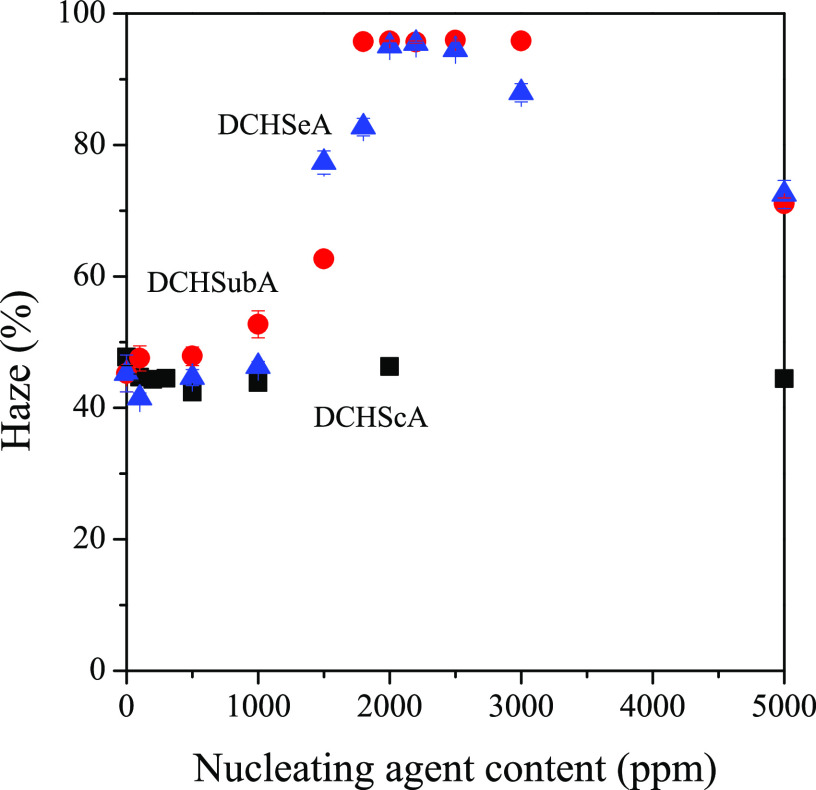
Haze value of samples nucleated with DCHScA (black square solid), DCHSubA (red circle solid), and DCHSeA (blue triangle up solid).

Optical properties of the nucleated samples are characterized with the value of haze. In Figure 19, we present haze as a function of DCHScA, DCHSubA, and DCHSeA contents. The presence of DCHScA (and also our other α-nucleating agents) has no significant effect on haze. This fact corroborates our previously mentioned idea, that the crystalline structure is neither microspherulitic nor microcrystalline, as in the case of these two, the value of haze decreases. In contrast, in the case of the other two nucleating agents, haze rapidly increases around the solubility limit of the nucleating agents, due to the appearance of β-iPP.

## 4 Conclusions

The aim of this work was to prepare and thoroughly study *N*,*N*′-dicyclohexylcarboxamide homologues from the viewpoint of applicability as nucleating agents in iPP. Seven of the nine compounds were successfully synthesized. One of the obtained materials (*N*,*N*′-dicyclohexyloxamide) cannot be used as a nucleating agent due to its low thermal stability. According to the qualitative investigation of nucleating efficiency and solubility, all synthesized additives were partially soluble in the polymer melt and proved to influence the crystalline structure developing in the polymer. The synthesized compounds have a nucleating effect in iPP; moreover, two of them (*N*,*N*′-dicyclohexylsuberoylamide and *N*,*N*′-dicyclohexylsebacoylamide) proved to be nonselective β-nucleating agents. This manifests itself also in the properties important from the viewpoint of applicability, as impact resistance and haze increase in a certain concentration range above the solubility limit of these new β-nucleating agents. According to the microscopic studies, a unique, dendritic crystalline structure develops in the polymer, which together with the presence of β-iPP results in improved impact resistance and higher opacity.

## Supporting Information Available

^13^C NMR spectra, FTIR spectra, and STA curves of the compounds investigated in the study (PDF)
