# Supplementary material for: Effect of N,N′-Dicyclohexyldicarboxamide Homologues on the Crystallization and Properties of Isotactic Polypropylene
Source: ACS Omega. 2021 Mar 23;6(13):9053–65. doi: 10.1021/acsomega.1c00064 (PMC8028137; doi:10.1021/acsomega.1c00064)
Supplement: Supplementary file 1 — ao1c00064_si_001.pdf [file ao1c00064_si_001.pdf]

Supporting information

Effect of *N,N'*-dicyclohexyldicarboxamide homologues on the crystallization and properties of isotactic polypropylene

FLÓRA HORVÁTH[1], LEVENTE BIHARI[1], DOMINIKA BODROGI[1], TIBOR GOMBÁR[1], BENDEGÚZ HILT[1], BALÁZS KESZEI[1], TAMÁS KRAIN[1], ANDRÁS SIMON[2], ALFRÉD MENYHÁRD[1]*

[1]Budapest University of Technology and Economics, Faculty of Chemical Technology and Biotechnology, Department of Physical Chemistry and Materials Science, Műegyetem rkp. 3., Budapest, HU 1111.

[2]Budapest University of Technology and Economics, Faculty of Chemical Technology and Biotechnology, Department of Inorganic and Analytical Chemistry, Szt. Gellért tér 4., Budapest HU 1111.

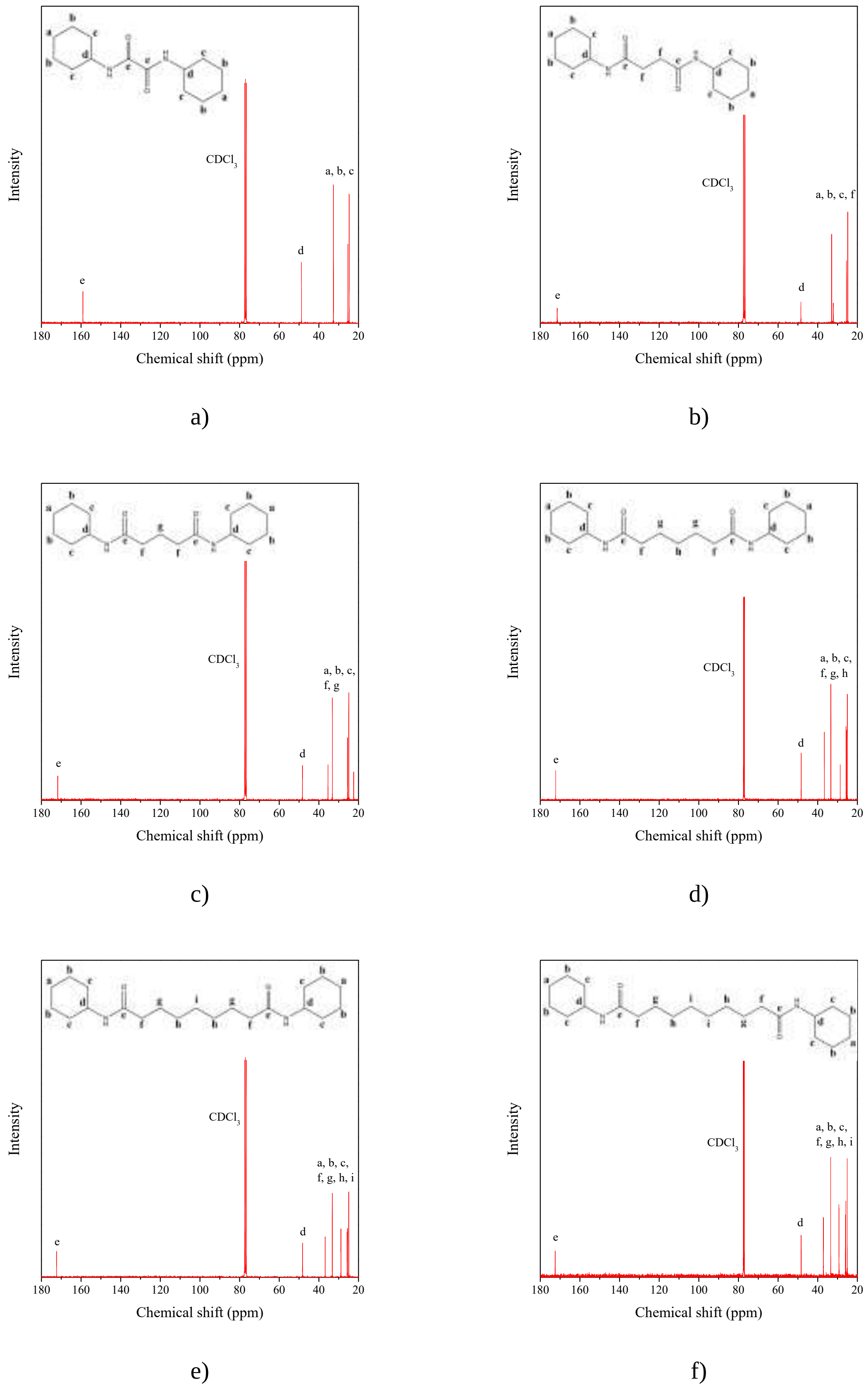

Figure S1 ^{13}C-NMR spectra of a) DCHOxA, b) DCHScA, c) DCHGlA, d) DCHPiA, e) DCHAzA, f) DCHSeA.

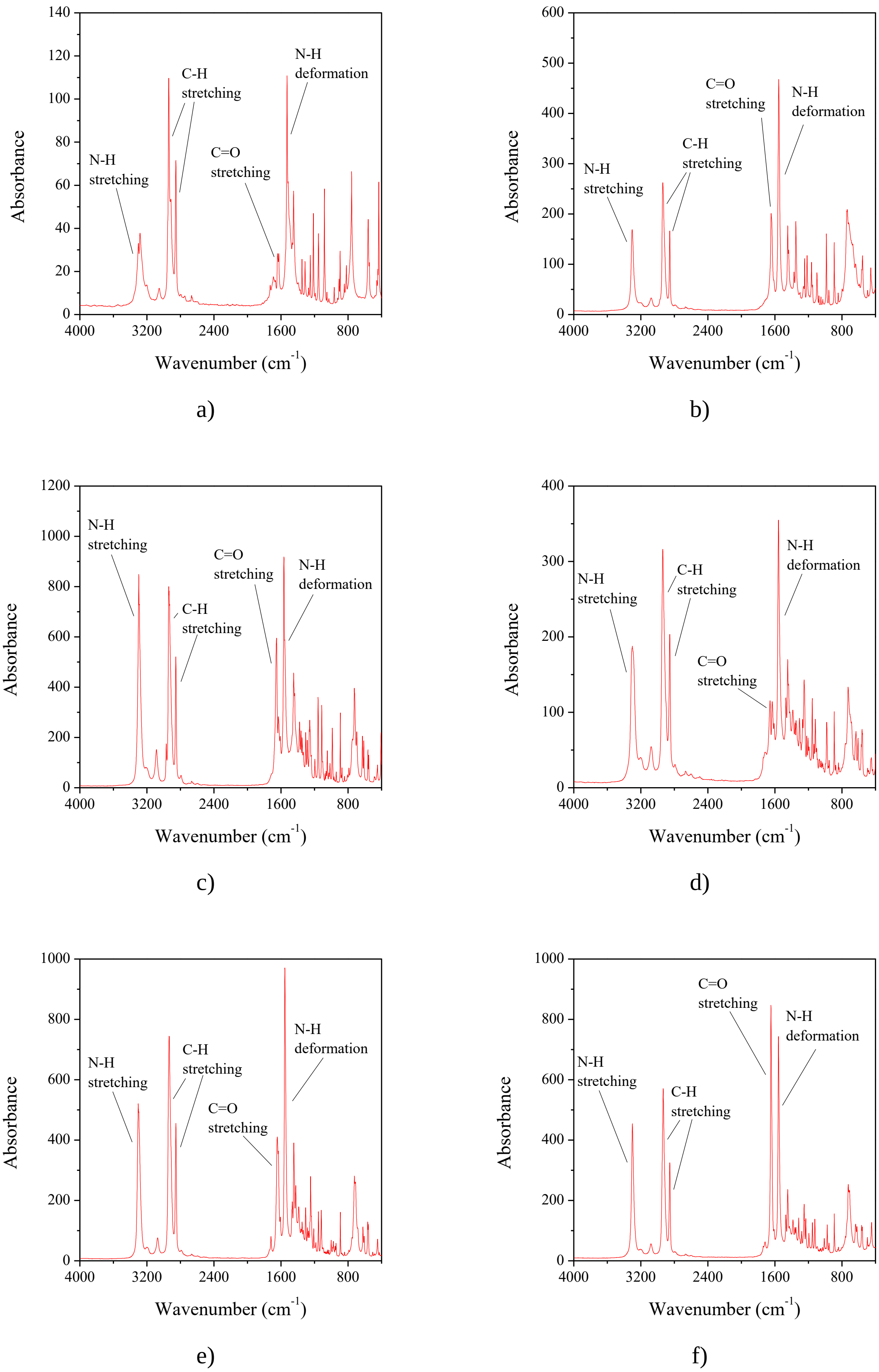

Figure S2 FT-IR spectra of a) DCHOxA, b) DCHScA, c) DCHGlA, d) DCHPiA, e) DCHAzA, f) DCHSeA.

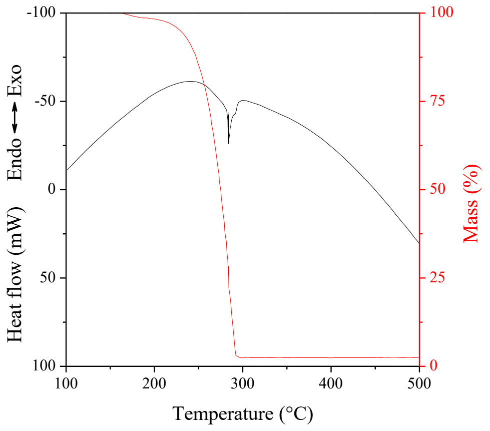

a)

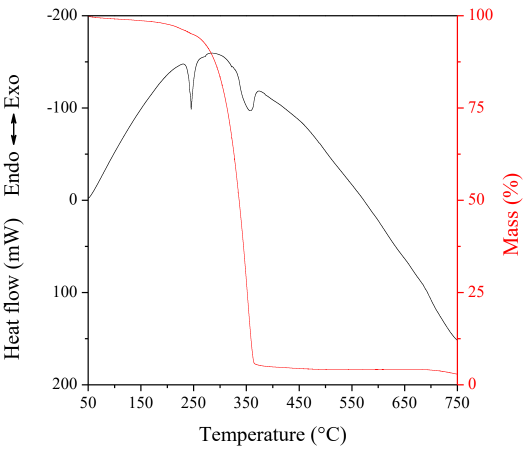

b)

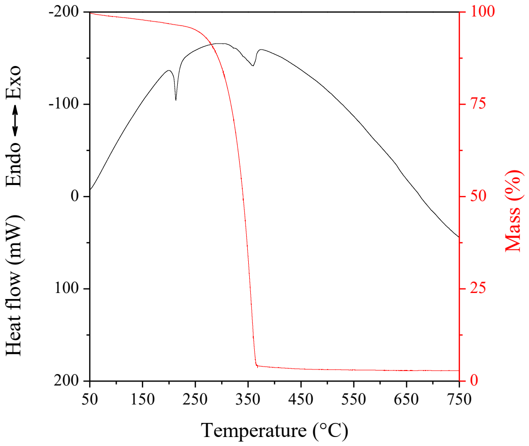

c)

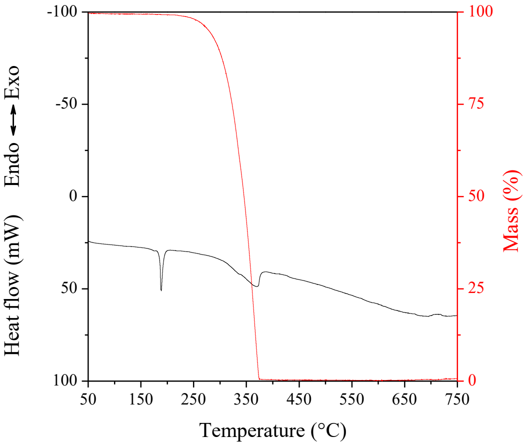

d)

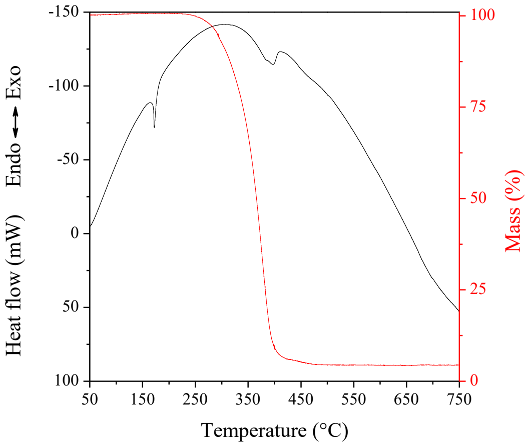

e)

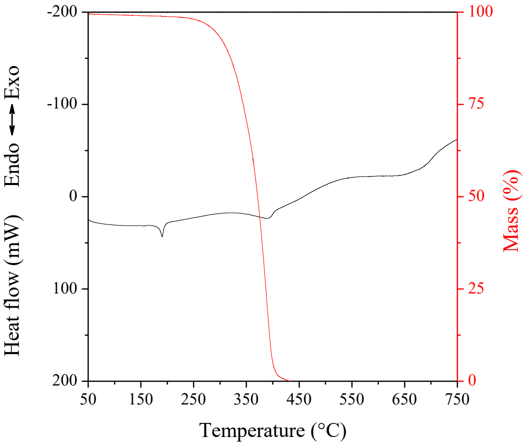

f)

Figure S3 Thermal stability and behavior of a) DCHOxA, b) DCHScA, c) DCHGlA, d) DCHPiA, e) DCHAzA, f) DCHSeA.
